# Methodological Considerations for a Diabetes Family-Based eHealth Intervention

**DOI:** 10.2196/48652

**Published:** 2023-09-18

**Authors:** Jan Brož, Matthew D Campbell, Pavlína Krollová, Juraj Michalec

**Affiliations:** 1 Department of Internal Medicine Second Faculty of Medicine Charles University Prague Czech Republic; 2 School of Nursing and Health Sciences University of Sunderland Sunderland United Kingdom; 3 Wellcome-MRC Institute of Metabolic Science University of Cambridge Cambridge United Kingdom; 4 Leeds Institute of Cardiovascular and Medicine University of Leeds Leeds United Kingdom

**Keywords:** public health, type 2 diabetes mellitus, intervention, randomized controlled trial, community health center

We read with interest the article by Feng et al [1], “The Effectiveness of an eHealth Family-Based Intervention Program in Patients With Uncontrolled Type 2 Diabetes Mellitus (T2DM) in the Community Via WeChat: Randomized Controlled Trial,” published in this journal.

This trial aimed to assess the effectiveness of an eHealth family-based health education intervention for patients with T2DM to improve their glucose control, risk perception, and self-care behaviors.

After 1 year of intervention, patients in the intervention arm showed significantly lower hemoglobin A_1c_ (HbA_1c_) values and improved several diabetes control–related skills (eg, general diet, special diet, blood sugar testing, foot care, risk knowledge, and personal control).

The authors concluded that the eHealth family-based intervention improved glucose control and self-care activities among patients with T2DM by aiding the implementation of interventions to enhance T2DM risk perceptions among family members. The intervention is generalizable for patients with T2DM using health management systems in community health centers. We applaud the authors in the preparation and execution of the study but have several questions that we feel would benefit the article’s readership.

In our observational study focused on metabolic control in patients with type 1 and type 2 diabetes treated with insulin in the Czech Republic and the Slovak Republic, we found a high clinical inertia resulting in a minimal and clinically insignificant difference in the mean HbA_1c_ within 3 years. Thus, we believe any new intervention targeting a long-term stabilized balance between the health carers’ therapeutic approach and the corresponding patient response is essential and can lead to substantial positive results [2,3].

Based on the 7-point scale, the differences in the individual skills observed are not very large. From the authors’ perspective, which intervention had the most significant effect on changes in HbA_1c_?

Patients in the intervention group met with their physicians every 3 months. Is information available on the frequency of doctor visits in the control group?

The improvements in HbA_1c_ values in the intervention arm are substantial. They even correspond to possible major changes in treatment (eg, initiation of insulin therapy) [4]. Were treatment changes monitored during the study, as these could be responsible for the HbA_1c_ improvement?

It is also known that patients with higher HbA_1c_ levels benefit more from changes in therapy [4]. A subanalysis of HbA_1c_ changes in correlation with their baseline levels would further contribute to the discussion of the generalizability of the intervention design.

We respectfully suggest considering these remarks, especially if a study continuation is planned.

## Abbreviations

HbA_1c_: hemoglobin A_1c_
T2DM: type 2 diabetes mellitus
